# Supplementary material for: Towards unified AI-driven fracture mechanics: the extended deep energy method (XDEM)
Source: Nat Commun. 2026 Aug 15;17:8492. doi: 10.1038/s41467-026-76748-1 (PMC13484471; doi:10.1038/s41467-026-76748-1)
Supplement: Supplementary file 3 [file 41467_2026_76748_MOESM3_ESM.zip › pic/Results/Stress_intensity_factor/Mixed/different_beta_contourf.pdf]

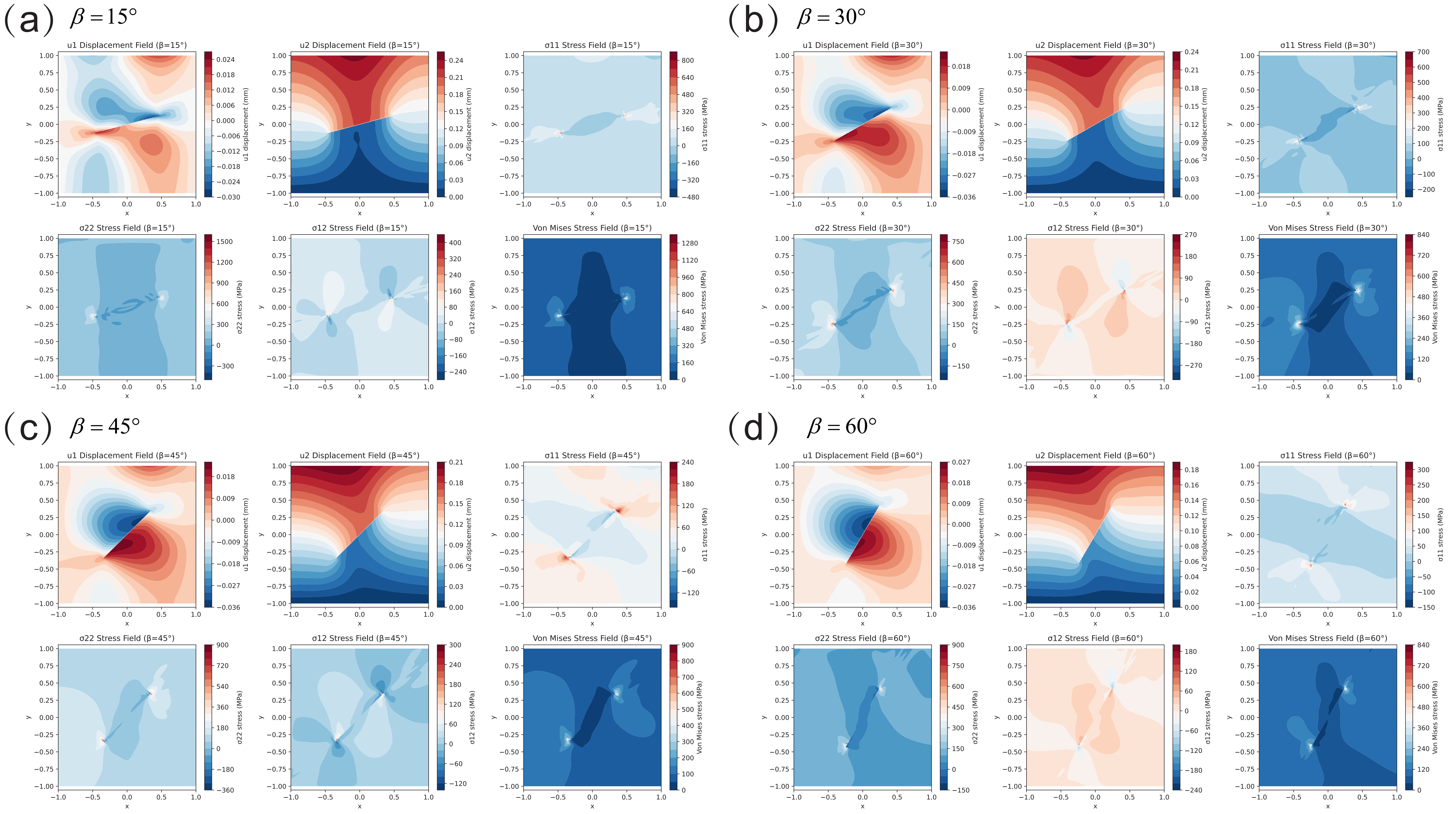
(a) $\beta = 15°$
u1 Displacement Field (β=15°)
u2 Displacement Field (β=15°)
σ11 Stress Field (β=15°)
σ22 Stress Field (β=15°)
σ12 Stress Field (β=15°)
Von Mises Stress Field (β=15°)
(b) $\beta = 30°$
u1 Displacement Field (β=30°)
u2 Displacement Field (β=30°)
σ11 Stress Field (β=30°)
σ22 Stress Field (β=30°)
σ12 Stress Field (β=30°)
Von Mises Stress Field (β=30°)
(c) $\beta = 45°$
u1 Displacement Field (β=45°)
u2 Displacement Field (β=45°)
σ11 Stress Field (β=45°)
σ22 Stress Field (β=45°)
σ12 Stress Field (β=45°)
Von Mises Stress Field (β=45°)
(d) $\beta = 60°$
u1 Displacement Field (β=60°)
u2 Displacement Field (β=60°)
σ11 Stress Field (β=60°)
σ22 Stress Field (β=60°)
σ12 Stress Field (β=60°)
Von Mises Stress Field (β=60°)
x
y
u1 displacement (mm)
u2 displacement (mm)
σ11 stress (MPa)
σ22 stress (MPa)
σ12 stress (MPa)
Von Mises stress (MPa)
